# Effects of Breast Surgery on Interoceptive Awareness in Females

**DOI:** 10.1093/asjof/ojae047

**Published:** 2024-07-02

**Authors:** Lauren E Weis, Haris M Akhter, Heidi H Hon, Perry J Johnson, Sean C Figy

## Abstract

**Background:**

The driving force for many seeking plastic surgery is comfort in one's body. Along with comfort come satisfaction, improved self-awareness, and potential change in interoceptive awareness—a term defined as the conscious perception of one's body. Although conscious perception of bodily signals is influenced by many factors, sense of self and body image play significant roles. Studies show diminished interoceptive awareness in those with negative body image, but no research has assessed the impact of change in body image on interoceptive awareness.

**Objectives:**

The purpose of this study is to investigate how interoceptive awareness changes following elective breast surgery.

**Methods:**

The Multidimensional Assessment of Interoceptive Awareness Version 2 (MAIA-2) was administered to females undergoing breast surgery. A baseline survey was administered preoperatively, with follow-up surveys at 1 week, 1 month, and 3 months postoperatively.

**Results:**

Data were collected from 39 females and analyzed using paired *t*-tests to compare MAIA-2 overall and subscores over time. Significance was seen at 1 week for subcategories of “not distracting” and “trust,” at 1 month for “trust,” and 3 months for “not worrying,” “emotional awareness,” “self-regulation,” and “trust.” Overall survey averages were significantly increased at all postoperative intervals.

**Conclusions:**

From this study, it can be concluded that breast surgery positively impacts interoceptive awareness. These findings are clinically relevant as they offer providers’ insight into the psychological effects of breast procedures. A comprehensive understanding of procedure outcomes enables providers to educate patients on both anticipated physical results and changes in sense of self.

**Level of Evidence: 2:**

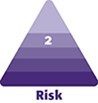

In the most simple terms, interoception is “the sense of the physiological condition of the body.^1^” Signals are relayed within the body and integrated to form a comprehensive sense of the body's current condition.^2^ Interoceptive *awareness* is a term used to define the conscious aspect of interoception.^1^ These conscious signals form one's internal state, which determines how one feels within their own body, including comfort, reliability, and overall well-being. As this is a very intricate process, there are many factors that may affect one's ability to accurately interpret these signals. If signals are not sensed or interpretated inaccurately, one's total perception of their body may be altered or compromised.

In addition to the aforementioned physiological signaling, psychological components strongly contribute to one's experience of self, with body image being an integral factor.^3^” Although body image may typically be thought to be rooted in physicality, interoception is a key component to one's body experience and thus body image.^4^ One's subjective image of themselves can alter reality based on preconceived thoughts and expectations.^5^ These preexisting thoughts about one's body can alter interpretation of physiological cues. Therefore, there is an intricate interplay between physiology and psychology influencing interoceptive awareness.

The relationship between body image and interoception has been explored in previous literature, although the population has been largely limited to those with eating disorders. It has been found that a negative body image has a positive correlation with interoceptive awareness.^6,7^ Although limited, research also show congruent findings in those without eating disorders.^8,9^ Aside from eating disorders, dysfunction in interoception has been linked to many mental health disorders including but not limited to anxiety, depression, and post traumatic stress disorder.^2^

Plastic surgery is a major vehicle by which people have the ability to change their physical appearance, and thus body image. Whether it be breast augmentation, gender affirming surgery, or reconstruction of a cleft palate, plastic surgery is known to have a drastic impact on both one's physical appearance, and as a result, a change in body image. Many studies have shown that individuals have a significantly more positive body image postoperatively.^10,11^ However, the full effect of plastic surgery on patients’ psyche and emotional state is not fully understood, nor will it likely ever be completely. One particular area of interest is the relationship between interoceptive awareness, body image, and plastic surgery. As detailed earlier, plastic surgery is known to alter body image, but the effects on interoception are unknown. Therefore, this study aims to assess interoceptive awareness over a period of time in which females undergo a procedure that alters body image, specifically breast surgery. By understanding this relationship, there will be better knowledge of the psychological and physiological effects of breast surgery, which can better guide patients in preparing for the outcomes of surgery.

## METHODS

The study design was submitted to and approved by the University of Nebraska Medical Center's Institutional Review Board (IRB# 0301-22-EP). Females undergoing breast surgery at a single institution from June 2022 to June 2023 were recruited to participate in this study based on the following inclusion criteria: age ≥19 years old, cis-gender, and either undergoing augmentation, mastopexy, or reduction procedure. Exclusion criteria included transgender females, those under the age of 19, and breast procedure related to malignancy reconstruction. Females who met these criteria were consented through phone, and electronic consents were emailed to be filled out and stored through Research Electronic Data Capture (REDCap). REDCap serves as a secure, web-based software platform to collect and store data. After data points and informed consent were submitted to REDCap, participants were sent the first survey form to be completed preoperatively. Each consecutive survey was completed, and the data were stored in a similar manner. This same process of electronic survey distribution and data collection was repeated at 1 week, 1 month, and 3 months postoperatively. If not completed, patients were sent automated reminder emails, with a follow-up call from research personnel 5 days later if not completed.

The survey employed in this study was the Multi-dimensional Assessment of Interoceptive Awareness 2 (MAIA-2), which served as an objective scoring instrument.^11,12^ This survey consists of 37 questions regarding multiple dimensions of interoceptive awareness and one's bodily experience. Each question is scored on a scale of 0 to 5, with 0 being “never” and 5 being “always.” Overall survey averages are able to be generated based on total scoring as well as averages for 8 subcategories: “noticing,” “not distracting,” “not worrying,” “attention regulation,” “emotional awareness,” “self-regulation,” and “trusting.” Although the MAIA-2 has been most commonly used in those with eating disorders and distorted body images, it has not yet been used in those undergoing a procedure known to change body image.

Overall MAIA-2 scores were collected for each interval, as well as each of the 8 subcategories. All scores were statistically analyzed with IBM SPSS using a paired *t*-test. A paired *t*-test was chosen as the dependent variables were quantitative (score on MAIA-2), as well as participants serving as their own controls, making the data dependent. An exact 2-tailed *P*-value was calculated using a significance value of <.05. Of note, in calculating averages and significance, values only include those reported by participants, with those lost to follow-up excluded. In addition, each average at postoperative intervals was compared with the preoperative average of that exact population. For example, the 3-month postoperative overall survey average was compared with the preoperative average of only those participants who completed the 3 months. Those lost to follow-up after 1 week or 1 month were not included in the preoperative average for the 3-month analysis.

## RESULTS

A total of 43 females consented to being in the study. One female did not complete the preoperative MAIA-2 and 4 females did not complete surveys following the preoperative survey, so they were excluded from the study. Therefore, data from 39 females were included in the study: 24 females completed the entire 3-month series of surveys, 7 females completed surveys through 1 month, and 8 females completed the surveys through 1 week. Females who only completed the preoperative survey were not included in the study results due to the within-subject design. The demographics of participants are summarized in Table 1. Of note, the vast majority of participants were undergoing breast reductions (23 females, 59%). The average age was 42 years with a range of 20 to 70. The full breakdown of procedures is summarized in Table 1.

**Table 1.** Demographics

Demographics	
Age	Mean: 42 (SD: 13.93) Range: 20-70
Procedure type	Reduction: 23 (59%)
	Augmentation + Mastopexy: 4 (10%)
	Augmentation + Mastopexy + Abdominoplasty: 2 (5%)
	Reduction + Abdominoplasty: 1 (2.5%)
	Capsulectomy + Implant exchange: 1 (2.5%)
	Implant removal + Mastopexy + Liposuction: 1 (2.5%)
	Implant removal: 1 (2.5%)

SD, standard deviation.

**Figure 1.**
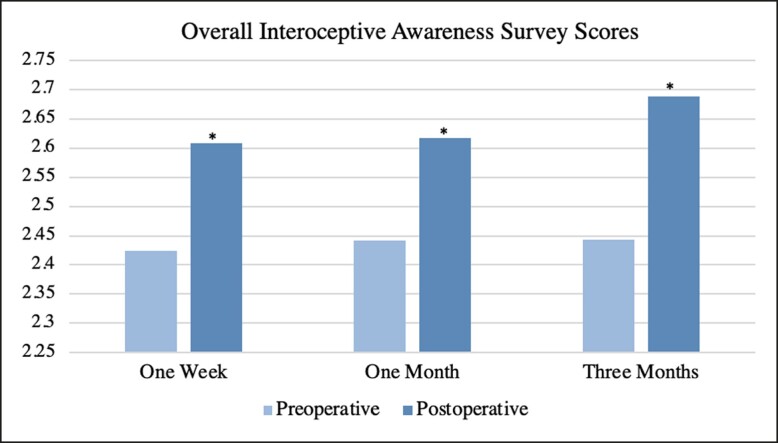
Overall interoceptive awareness survey averages over study duration. Each postoperative average is compared with the preoperative average of its respective population. Significance is indicated by asterisk.

For overall survey averages, there was a significant increase at all 3 postoperative time intervals, with *P*-values being .037 at 1 week, .021 at 1 month, and .008 at 3 months. The mean overall MAIA-2 survey scores and respective *P*-values are summarized in Table 2, with a visual representation displayed as Figure 1.

**Table 2.** Overall Interoceptive Awareness Scores

	Overall survey average	*P*-value
Preoperative, *n* = 39	2.425	—
Postoperative 1 week	2.608	.037^a^
Preoperative, *n* = 31	2.425	—
Postoperative 1 month	2.617	.021^a^
Preoperative, *n* = 24	2.435	—
Postoperative 3 months	2.688	.008^a^

^a^Significant.

**Figure 2.**
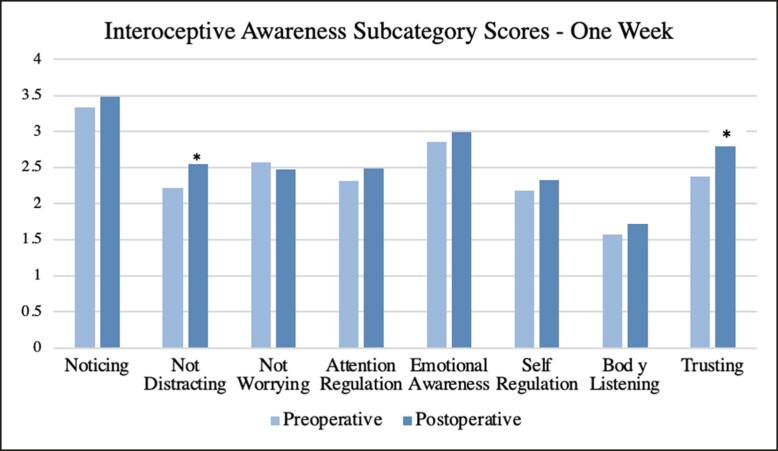
The interoceptive awareness subcategory averages at 1 week postoperative. Each postoperative average is compared with the preoperative average of its respective population. Asterisk indicates significance.

**Figure 3.**
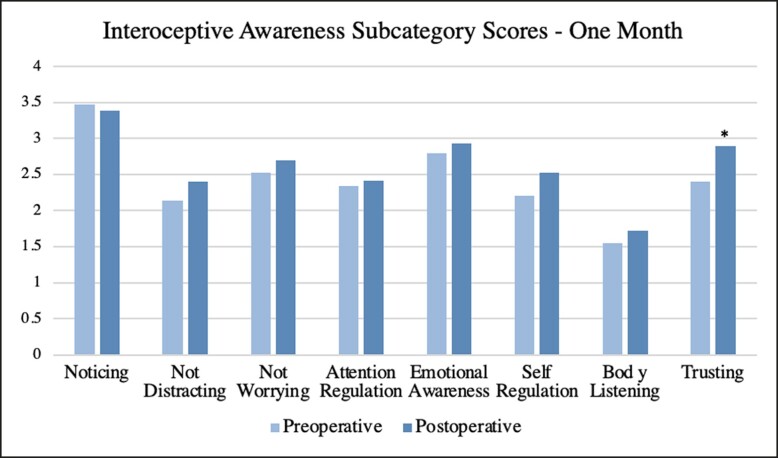
The interoceptive awareness subcategory averages at 1 month postoperative. Each postoperative average is compared with the preoperative average of its respective population. Asterisk indicates significance.

**Figure 4.**
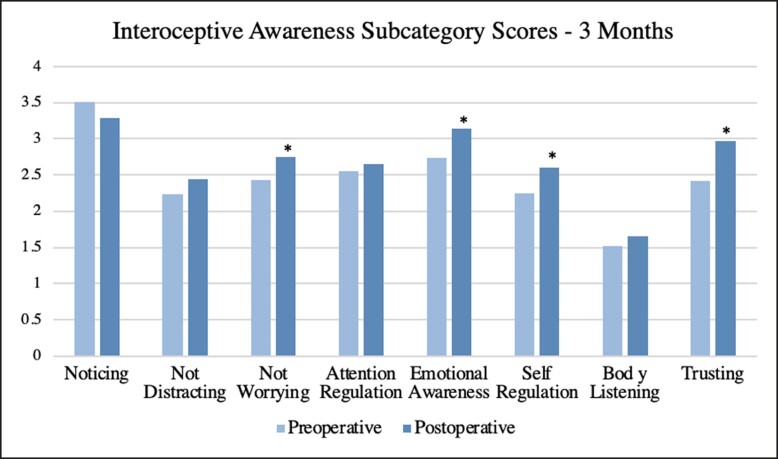
The interoceptive awareness subcategory averages at 3 months postoperative. Each postoperative average is compared with the preoperative average of its respective population. Asterisk indicates significance.

Surveys were further broken down into subcategories and averages were obtained along with *P*-values for each postoperative interval. At 1 week, a significance was found for the subcategories of “not distracting” (*P* = .040) and “trusting” (*P* = .019). At 1 month, there was a significant increase in the subcategory of “trusting” (*P* = .004). At 3 months, there was a significant increase in subcategories of “not worrying” (*P* = .026), “emotional awareness” (*P* = .006), “self-regulation” (*P* = .022), and “trusting” (*P* = .008). No significant trends were seen for the subcategories of “noticing,” “attention regulation,” and “body listening.” A complete list of subcategory averages and *P*-values is summarized in Table 3. A visual representation of subcategory averages broken down by time intervals is shown in Figures 2-4.

**Table 3.** Interoceptive Awareness Subcategory

Mean scores with *P*-value						
Subcategory	Time interval					
	Preoperative, *n* = 39	1-Week postoperative	Preoperative, *n* = 31	1-Month postoperative	Preoperative, *n* = 24	3 Months postoperative
Noticing	3.3289	3.4868 (0.294)	3.4677	3.3871 (0.625)	3.5104	3.2917 (0.214)
Not distracting	2.2193	2.5439 (0.040)^a^	2.1452	2.3978 (0.120)	2.2292	2.4444 (0.099)
Not worrying	2.5684	2.4737 (0.371)	2.5161	2.6903 (0.174)	2.4333	2.7417 (0.026)^a^
Attention regulation	2.3158	2.4850 (0.161)	2.3318	2.4055 (0.604)	2.5476	2.6548 (0.458)
Emotional awareness	2.8579	2.9842 (0.363)	2.7871	2.9290 (0.247)	2.7333	3.1417 (0.006)^a^
Self-regulation	2.1711	2.3224 (0.244)	2.2016	2.5161 (0.056)	2.2396	2.6042 (0.022)^a^
Body listening	1.5724	1.7171 (0.281)	1.5484	1.7177 (0.137)	1.5208	1.6563 (0.425)
Trusting	2.3684	2.8509 (0.019)**^a^**	2.3978	2.8925 (0.004)^a^	2.4167	2.9722 (0.008)^a^

^a^Significant.

## DISCUSSION

Elective breast surgery had a positive impact on interoceptive awareness as early as 1 week postoperative, with sustained improvement up to 3 months postoperative in females. The psychological benefit was seen before physical healing was completed, which may indicate that the mere knowledge of having breast surgery relieves some internal stress and has a direct effect on the psyche, rather than secondary to physical appearance. These findings expand on current literature regarding the understanding of interoceptive awareness and the many factors that influence it. As mentioned previously, little is known about the relationship between body image and interoceptive awareness, and even less about how changes in body image impact interoceptive awareness. This study provides insight into that intricate relationship.

In discussing individual subcategorical results, it is imperative to understand the definition of each in the setting of interoceptive awareness. At 1 week, there was a significant increase in “not distracting” and “trusting,” defined by the “tendency not to ignore or distract oneself from sensations of pain or discomfort” and “experience of one's body as safe and trustworthy,” respectively.^12^ Although an increase in “not distracting” at 1-week postoperation may be secondary to increased or new pain, it can also be due to the psychological effects of surgery as well. Prior to surgery, females may be used to feeling uncomfortable in their bodies either secondary to body image or direct physical pain of macromastia, for example. Following surgery, they may expect to have those feelings diminished or resolved. So, when feeling discomfort or pain, they may be more sensitive or responsive. As for “trusting,” it is significant that despite being wrapped in bandages, changing dressings, and with a possible drain in place, females feel their body is safer than it was preoperatively. At 1 month and 3 months, this upward trend in “trusting” persists, showing that as females spend more time in their new body, they continue to feel safer and rely upon their own body.

In addition to “trusting,” a significant increase at 3 months was seen in the subcategories of “not worrying,” “emotional awareness,” and “self-regulation.” “Not worrying” is defined as a “tendency not to worry or experience emotional distress with sensations of pain or discomfort.”^12^ This finding may correlate with the increase in “trusting”—as females trust their bodies more, they are no longer as concerned with dysphoric feelings. This does not mean that they do not pay attention to these feelings (not distracting), but rather do not become overly concerned with them. “Emotional awareness” is defined as “awareness of the connection between body sensations and emotional states”.^12^ Preoperatively, this study found that “emotional awareness” was lower when compared with postoperative states, representing that preoperatively, there is dysfunction at some level in the emotional processing of bodily sensations. This can be due to changes in neural firing from either a bottom-up approach, or a top-down approach, controlled by higher functioning areas in the cortices. The latter is more likely, as body image is a perceived notion that is being altered in this study, leading to the influence of the interpretation of basic signals. Lastly, “self-regulation” is the “ability to regulate distress by attention to body sensations.”^12^ This is quite closely related to “not worrying.” Although “not worrying” is related to not *experiencing* emotional distress when feeling discomfort, “self-regulation” is the ability to regulate this distress when the feeling arises. This change in body image following breast surgery may create a sense of well-being and comfort in one's body, thereby diminishing anxiety provoked by discomfort and feeling a sense of security when it does arise.

No significant increase was seen in the subcategories of “noticing,” “attention regulation,” or “body listening.” “Noticing” refers to “awareness of uncomfortable, comfortable, and neutral body sensations.” Interestingly, although there was an insignificant increase at 1 week, there was an insignificant decrease at 1 month and 3 months postoperation. In the perioperative period, patients experienced an abundance of new sensations and feelings, which may overwhelm their ability to pick up on all sensations. This may take months to years to reverse, but it may be improved by implementing interoceptive awareness improving measures, such as mindful awareness in body-oriented therapy (MABT).^13^ “Body listening,” defined as “active listening to the body for insight,” may also be diminished for the same reason and may take years to improve upon. Although not significant, an increase was seen in this subcategory at all postoperative time intervals, exemplifying the power of breast surgery early on. Lastly, “attention regulation” is the “ability to sustain and control attention to bodily sensations.”^12^ This too could be improved upon by implementing MABT.

There are limitations involved in this study, the first being the inherit limitation of using a self-reported survey. Patients may respond in a way they feel they should, regardless of their true feelings. They may also be influenced by availability heuristics and respond accordingly, rather than their general feelings in daily life (per survey instructions). Secondly, results may be influenced by the type of breast surgery. The majority of the participants underwent breast reductions, which may generate different effects when compared with augmentations or mastopexies. This may skew data to be more or less significant than reported. Another factor that may influence results is the involvement of additional procedures and complications. Many of the females included in the study also underwent liposuction or abdominoplasties, which eliminates the ability to directly correlate breast surgery with outcomes. Complications were not directly reported and thus not analyzed, although may impact data. Finally, this study only followed females out to 3 months postoperative. Some results may take months to see effects and were thus not observed.

For future research endeavors, results may be stratified based on breast procedure type, rather than grouping all participants together. This would allow us to delineate the effects of specific procedures on interoceptive awareness. This would require a larger sample size, as the population of females undergoing augmentations is relatively small. Results may also be stratified based on indication for surgery—whether it be for purely aesthetic purposes vs medical necessity (relieving back pain, shoulder strain, etc). Those with the desire for a change in body image may have different outcomes than those whose primary intention is to relieve discomfort or pain. This study focused only on breast procedures, but combination procedures are common and often included. For example, this study included a patient undergoing an abdominoplasty as well as breast surgery, which could affect interoceptive awareness independently. Additive effects may be seen in these patients, but further research is needed to isolate the procedures and work to understand the relationship between combination procedures and interoceptive awareness.

Further variables of interest include age and history of mental health diagnoses. Females of different ages may have different perceptions and expectations of their body, which may influence their experience with them and how they score the MAIA-2. Mental health diagnoses also play a significant role. As mentioned earlier, eating disorders have been shown to have a significant independent effect on body image.^6,7,9,14^ Even more common are depression and anxiety, which were not included as variables in this study. Additional research is needed to understand the effects of these psychological conditions on body image as a whole, as well as when undergoing plastic surgery.

Clinically, these findings benefit both the surgeon and the patient. With this knowledge, providers are better suited to educate their patients on the potential psychological outcomes of plastic surgery, in addition to the anticipated physical outcome. A significant portion of recovery is becoming comfortable in one's “new” body, and any advice on what to expect will be of use to patients. Additionally, this information may assist surgeons in planning procedures for patients based on their goals of surgery.

## CONCLUSIONS

Elective breast surgery has a significant positive impact on interoceptive awareness in the first 3 postoperative months. These findings demonstrate the impact of breast surgery on the mind–body interaction and how it increases sensitivity to physiological stimuli, ability to integrate, and accuracy of interpretation. Findings such as these are imperative to fully elucidating the relationship between breast surgery and psychological outcomes. A holistic approach to the anticipated outcomes allows providers to fully educate and prepare patients to make the most appropriate surgical decision for themselves—both for their body and mind.
